# The relationship between parental socio-economic status and episodes of drunkenness among adolescents: findings from a cross-national survey

**DOI:** 10.1186/1471-2458-6-289

**Published:** 2006-11-28

**Authors:** Matthias Richter, Anja Leppin, Saoirse Nic Gabhainn

**Affiliations:** 1 School of Public Heath, University of Bielefeld, Universitätsstr. 25, 33613 Bielefeld, Germany; 2 Unit for Health Promotion Research, University of Southern Denmark, Niels Bohrs Vej 9, 6700 Esbjerg, Denmark; 3 Department of Health Promotion, National University of Ireland, Galway, Ireland

## Abstract

**Background:**

Behavioral factors such as (excessive) alcohol consumption play a major role in the explanation of social inequalities in health. The unequal distribution of health risk behaviors among socio-economic groups has important consequences for both the current and future health status of the younger generation. However, little is known about socio-economic differences in unhealthy lifestyles during adolescence. The purpose of the present study is to investigate socio-economic differences in adolescent drinking behaviour among 11–15 year old adolescents in Europe and North America.

**Methods:**

Data was obtained from the Health Behaviour in School-aged Children (HBSC) study 2001/02, a cross-national survey conducted in collaboration with the World Health Organization. The present analysis is based on 69249 male and 73619 female students from 28 countries. The effect of parental occupation and family affluence on episodes of drunkenness was assessed using separate logistic regression models controlling for age.

**Results:**

Socio-economic circumstances of the family had only a limited effect on repeated drunkenness in adolescence. For girls only in one out of 28 countries a significant association between family affluence and repeated drunkenness was observed, while boys from low and/or medium affluent families in nine countries faced a lower risk of drunkenness than boys from more affluent families. Regarding parental occupation, significant differences in episodes of drunkenness were found in nine countries for boys and in six countries for girls. Compared to family affluence, which was positively related to risk of drunkenness, a decreasing occupational status predicted an increasing risk of drunkenness. This pattern was identified within a number of countries, most noticeably for boys.

**Conclusion:**

Parental socio-economic status is only of limited importance for episodes of drunkenness in early adolescence, and this very limited role seems to apply for girls more than for boys and for parental occupation more than family affluence. For future studies it might be important to look at the effects of socio-economic status within the context of other peer, family and school related factors in order to assess to what extent those factors might mediate the effects of social class background.

## Background

Numerous studies have shown that socio-economic status (SES) has a profound influence on mortality and morbidity in adulthood [1-3]. Behavioral factors such as smoking, malnutrition, excessive consumption of alcohol and lack of physical activity play a major role in the explanation of these social inequalities in health [4,5].

Although the importance of individual lifestyle patterns for current and future health has long been accepted, little is known about the extent and pattern of socio-economic differences in health behavior during adolescence. Despite the relative consistency of socio-economic differences in health risk behavior in adulthood [6-9], it is less clear at which stage in life these differences are established [10]. In addition, the existing evidence about the relationship between socio-economic status and health risk behaviors in adolescence is inconsistent and even contradictory. This is especially true for adolescent drinking behavior. While some studies have identified a higher risk of excessive adolescent drinking behavior among lower socio-economic groups [11-13], others have found no or even inverse social gradients in adolescent alcohol consumption [14-20]. To our knowledge, the present analysis is the first to examine the influence of socio-economic status on drunkenness among adolescents in a wide range of European and North American countries.

The study will address the following research questions: (1) whether socio-economic differences in episodes of drunkenness exist among 11–15 year old adolescents in Europe and North America, (2) whether there are differential effects of SES in different geographical regions of Europe, and (3) whether these relationships change with type of SES indicator or gender.

## Methods

Data were obtained from the Health Behaviour in School-aged Children (HBSC) study 2001/2002, a World Health Organization collaborative cross-national survey [21]. The aim of the HBSC study is to describe young people's health and health behavior and to analyze how these outcomes are related to the social context. Cross-sectional surveys of 11-, 13- and 15-year-old children and adolescents are carried out every four years in a growing number of countries based on an internationally agreed protocol [22]. The latest survey, in 2001/02, included a total of 35 countries from Europe and North America. A detailed description of the aims and theoretical framework of the study has been described elsewhere [21,22].

### Sample

Students are selected using a clustered sampling design, where the initial sampling unit is the school class. Approximately 1500 respondents in each of the three age groups (i.e. 11, 13 and 15 year olds) were targeted in every country. A regional sample was selected in Germany (Nordrhein-Westfalen, Berlin, Hessen and Sachsen). Separate studies cover the Flemish and French speaking populations in Belgium and separate studies are also undertaken for England, Scotland and Wales in the UK. In some of the countries participating in the HBSC study ethical approval was not necessary or applicable at the time. In other countries that required approval this was obtained by different Institutional Review Boards (see additional file 1: Level of consent and ethical approval in HBSC.doc). Detailed documentation on the level of consent by school, parental and student level across countries is also given in additional file 1. The present analysis is based on 69249 male and 73619 female students from 28 countries. Countries excluded from the analysis were Austria, Belgium (Wallonia), England, Greenland, Lithuania, Macedonia and the Netherlands because of high rates of missing values for occupational status (above 15%).

### Instrument and Variables

The data were collected by means of standardized questionnaires, administered in school classrooms according to standard instructions. The questionnaire consisted of a number of mandatory questions, which were the same in all participating countries, and several optional items, which allowed participating countries to include additional questions of national interest.

#### Drunkenness

Frequency of drunkenness was assessed by asking whether the adolescents had ever had so much alcohol that they were really drunk. Possible answers were: "no, never", "yes, once", "yes, two to three times", "yes, four to ten times" and "yes, more than ten times". Response options were dichotomized into 1 = two to three times or more and 0 = less than two to three times.

#### Parental Occupation

Two open-ended questions assessed parents' occupational status. Students were asked to indicate separately where their father and mother worked and to describe what kind of job they did. Countries were required to condense the answers into a classification similar to the British Registrar General's social classification of occupations ranging from 1 (high occupational status) to 5 (low occupational status). Since many mothers were economically inactive and many responses on parental occupation were missing, information on the occupational status of the father and mother was combined, using the highest occupational status for each couple as the parental indicator. The child was grouped in the lowest occupational category, if neither parent was working for money ("looking for a job", "full time at home"). Adolescents who lived with a single parent were also grouped in the lowest category, when the single parent was "unemployed", "sick, retired, or a student" or "full time at home". In order to obtain three groups of similar size, the original five categories were recoded into high (1 and 2), middle (3) and low (4 and 5) occupational status. Table 1 presents the distribution of parental occupation and family affluence in the study population.

#### Family affluence

The "Family Affluence Scale (FAS)" was used as an alternative measure of parental socio-economic status [23-25]. The scale consists of four items: does your family own a car (0, 1, 2 or more), how many times did you travel away on holiday with your family during the past 12 months? (0, 1, 2, 3 or more), do you have your own bedroom for yourself? (0, 1), how many computers does your family own (0, 1, 2, 3 or more). A composite FAS score was calculated by summing the responses to these four items ranging from 0 to 9. The FAS scores were subsequently recoded into tertiles within each country (high, middle, low).

### Statistical Analyses

Logistic regression analyses including both SES indicators simultaneously were used to investigate their independent effect on episodes of drunkenness. Spearman's rho values for the relation between family affluence and parental occupation varied from 0.18 in the Ukraine to 0.38 in Germany. Using separate models for family affluence and parental occupation gave findings very similar to those of the combined model. All models were adjusted for age (dummy variable with three categories: 11, 13 and 15 year olds), because of its major relevance for occurrence of these lifestyle behaviors in adolescence. Results are presented as odds ratios with 95% confidence intervals. The highest group of family affluence or parental occupation respectively served as the reference category with odds ratios being computed for the other two groups in comparison. All analyses were conducted separately for each country and gender. To take into account the possible effect of cluster sampling, all reported odds ratios and 95% confidence intervals are based on robust standard errors. All analyses were done using Stata Statistical Package, version 8.2 (Stata Corporation, College Station, Tex).

## Results

### Family affluence and drunkenness in adolescence

Table 2 shows the relationship between repeated drunkenness and family affluence for 11 to 15 year olds across countries for boys and girls. After adjusting for age and each SES indicator respectively a significant effect for boys was found in nine countries for family affluence. For boys in Croatia, Hungary, Portugal, Russia and Sweden the risk of repeated episodes of drunkenness decreased with decreasing family affluence. In Scotland, Latvia, Estonia and Germany the lowest risk of repeated drunkenness was observed for boys from middle affluent families. For girls repeated drunkenness was found to be largely unrelated to socio-economic circumstances of the family. Only Latvian girls from low affluent families had a significant lower lifetime risk of being drunk 2–3 times or more.

### Parental occupation and drunkenness in adolescence

In terms of parental occupation a different pattern than for family affluence emerged for boys (Table 3). Of the nine countries that showed significant effects, the risk of drunkenness increased with decreasing occupational status in six countries (Croatia, Czech Republic, Hungary, Belgium, Russia and Slovenia). Three of these countries (Croatia, Hungary and Russia) showed a decreasing risk with decreasing family affluence and an increasing risk with decreasing occupational status. For the remaining countries (Finland, Sweden and Wales) the highest risk of repeated drunkenness was observed in the middle family affluence category followed by the lowest category. For girls a significant effect of parental occupation was observed in six countries. Here, the risk of repeated drunkenness increased with decreasing occupational status in Finland, Belgium and Norway, while for Ireland the highest odds ratio was found in the middle occupational status category. For French girls the direction of the effect was reversed, indicating a significantly lower odds ratio for repeated drunkenness for girls with a mid-range as compared to a high occupational status of their parents. A lower risk of being drunk 2–3 times in life was also found for girls from Poland but in this case the effect was observed in the lowest occupational status category.

## Discussion

Little is known about the relationship between socio-economic status and adolescent drinking behavior. While several studies have investigated the relationship between SES and various measures of drinking behavior in youth, findings have been very heterogeneous. The HBSC study provides a unique opportunity to allow for between-country comparisons, using the same data collection protocols.

Overall, we found only very limited evidence for a close consistent relationship between parental SES and episodes of drunkenness among 11 to 15 year olds. Our findings therefore underline previous studies which have identified weak or even no associations between parental SES and alcohol use in adolescence [20,16-18]. Some differences are noted, though, with regard to gender. For girls, almost no significant effects for family affluence and only few effects for parental occupation were observed. For boys, on the other hand, there was at least some evidence for socio-economic differences. Adjusted for parental occupation, it was found that boys from low and/or medium affluent families faced a lower risk of frequent drunkenness than boys from highly affluent families. While such effects occurred in one third of the countries, it should be noted that in the large majority of countries, trends for low affluent boys to be less at risk than high affluent boys became apparent, even if these did – sometimes barely – miss levels of significance. Thus, for boys at least, it might be argued that the data suggest an adolescent-emergent role of SES in some countries that only become manifest in adulthood.

Kuntsche et al. have pointed out that while for adults problem drinking seems to be more common in less affluent groups, this direction might be reversed for adolescents, where accessibility of financial resources is more limited [26]. If one reasonably assumes that availability of pocket money is at least to some degree related to parents' affluence, the results of two Finnish studies, which found a clear relationship between adolescents' own financial resources, i.e. amount of pocket money and drunkenness support this view [27,28].

As for geographical differences, it might seem difficult to identify clear patterns at a first glance. A closer look, however, reveals that it was particularly in countries with either an overall lower income level and a strong "alcohol culture" (according to overall consumption rates [29]), such as Eastern European countries like Croatia, Estonia, Hungary, Latvia, Russia or with particularly high alcohol prices such as in Sweden that effects of family affluence on drunkenness in boys became apparent. Countries with no SES effects seem to be predominantly those with either a high overall income level such as Switzerland and/or very strong "alcohol cultures" such as Denmark, Finland and France or particularly low alcohol prices, as in the case of Spain.

Regarding parental occupation there were also more effects on drunkenness for boys than for girls. Contrary to FAS, however, these effects indicated a higher risk in adolescents with low or medium occupational status parents. Thus, while for FAS the risk of repeated drunkenness decreased with family affluence, for parental occupation, the risk tended to increase with decreasing status, which is in line with some findings from other studies [14,15]. This was even true for countries that showed significant effects on both indicators. Given the positive relationship between parental occupation and family affluence this result might appear somewhat surprising. However, it should be acknowledged that the correlation between these two indicators is rather low. Therefore, these findings suggest that the different dimensions of socio-economic circumstances also have different effects on drunkenness in adolescence.

A possible explanation for these differences might be found when the specific consequences deriving from the two SES dimensions are considered. Parental occupation to some extent reflects parental education, and parents from a higher educational background might be somewhat more likely to influence substance use in adolescence by their parenting strategies and the values and norms they transmit to their offspring. Similarly, differential processes of modeling behavior might have an impact – at least as far as fathers are concerned. European comparison studies among adults have thus shown that heavy drinking episodes are more common among lower educational groups [30], particularly among men with lower educational levels, while for women no such differences appeared [31]. In an international comparative study Bloomfield et al. also reported no significant social inequalities in terms of educational level for binge drinking in women, but relatively consistent, although often not significant, gradients for males, with the lower and middle educational groups being more likely to be heavy episodic drinkers than the higher educated [32].

Family affluence, on the other hand, appears to be more strongly related to income or spending patterns, therefore indicating availability of resources to indulge in the relatively costly consumption of alcohol in the first place. This might also explain at least part of the gender differences found in the relationship between FAS and drinking behaviour. As the overall rate of repeated drunkenness is much higher in boys than in girls, the relative relevance of financial resources, such as pocket money to buy alcohol, might also be higher among boys, thus increasing the role of family affluence for this group.

In addition, there might be other factors during adolescence, which may have a greater impact on drinking behavior than parental SES. For instance, the specific character of adolescence as a stage of experimenting with 'new' behaviors associated with an adult status might also create generalized developmental stage-related demands and temptations that tend to transcend boundaries created by the family's socio-economic background. More important, yet, might be the consideration that while health behavior in childhood is strongly determined by parents, with the onset of adolescence the influence of peers and youth culture increases in relation to more traditional family norms and values, and this shift in influences is closely related to the emergence of adolescent risk behavior [33-35]. This might especially apply to those behaviors which do not commence until adolescence such as alcohol and tobacco consumption.

### Study Limitations and Strengths

Several methodological aspects restrict the explanatory power of these findings. A point which thus might be considered problematic is the usage of self-report data on parental occupation. Even though several studies indicate that the classifiable answers of adolescents (even at the age of 11 to 13) can be considered as good proxy reports of parental occupation [36-38], it remains problematic that a relatively large number of adolescents (up to 15%) do not seem to be willing or able to report their parents' occupation at all. If missing responses are unequally distributed among socio-economic groups, which might be expected, this may effect findings. Therefore, the results on occupational differences must be interpreted cautiously.

Similarly, assessing adolescent drinking behavior by means of self-report might be considered problematic. Self-reports of alcohol consumption and other substance use patterns, can not only incur general problems of social desirability, but might also result in different response tendencies according to the reference group or gender. However, it has been repeatedly shown that self-reports can usually claim a rather high degree of validity [39,40]. Moreover, in order to underline the importance of giving honest responses, the students in the present study were assured of confidentiality and that neither their parents nor teachers would be informed about the individual results. Another source of self-reporting bias would emerge if under-reporting was associated with parental SES. Unfortunately, not much is known about these mechanisms in adolescence, but if underreporting was unequally distributed across SES, then this may have biased the results reported here. Nevertheless, while the usage of a dichotomous classification of drunkenness might be considered a crude type of assessment, it is probably less vulnerable to such reporting errors.

Even though using the highest occupational status of the parents has the advantage of reducing the numbers of missing, it does not facilitate an assessment of gender influences in the association between the prevalence of repeated drunkenness and parental occupation. Therefore, the analysis was re-run using fathers' and mothers' occupational status separately (not shown). Overall, the results support the application of the head of the household measure (i.e. the highest occupational status of each couple) as among girls – in those few countries where an effect was found – both mothers' as well as fathers' occupational status were significantly related to drunkenness. Among boys, a rather inconsistent picture emerged. The occupational status of the father was associated with drunkenness in only three countries while the status of the mother was related to drunkenness in five countries. In addition, in five countries that showed significant associations with the head of the household measure, no effect for fathers' or mothers' occupation was found.

Another issue that should be considered is that by not adjusting the significance level for multiple testing the findings might have capitalized by chance. In fact we decided against such adjustment based on the rationale that the purpose of research here is more of an exploratory kind, so that the increased likelihood of a Type II error that results from reducing type I error by Bonferroni-adjustment might be considered just as committing a type I error. Also, one could argue that in fact the different samples we looked at came from different and separate country studies (even if they were based on the same study protocol) and there was no cross-sample testing but only separate tests within the different samples/studies are reported.

However, if such an adjustment was made this would indeed leave fewer significant findings, considering the number of countries involved, and result in more non-significant trends. Nevertheless, the results would not fundamentally alter the main findings of the study, i.e. that compared to adulthood parental SES is of minor importance in the prediction of episodes of drunkenness in adolescence, and that only in some countries and only for boys a pattern of socio-economic differences in drunkenness emerges.

## Conclusion

The present study clearly indicates that SES seems to be of limited importance for the development of excessive alcohol use in early adolescence, and this limited role seems to apply for girls more than for boys and for parental occupation more than for family affluence. However, it might be interesting for future studies to look at the effects of socio-economic status within the context of other, possibly more relevant, factors such as peer or school influence on one hand or other parental factors such as social support on the other in order to assess to what extent those factors, which are distinctive for adolescents, might strengthen or weaken the effects of socio-economic background.

## Competing interests

The authors declare that they have no competing interests.

## Authors' contributions

MR was responsible for originating the study, analyzing and interpreting the data, and writing the article. AL analyzed the data and contributed to writing the article. SNG assisted with writing and data interpretation and provided feedback on drafts. All authors helped to conceptualize ideas, interpret findings and review drafts of the article. All authors read and approved the final manuscript.

## Pre-publication history

The pre-publication history for this paper can be accessed here:



## Supplementary Material

Additional file 1 Appendix 1: Information on level of consent and ethical approval in the HBSC study 2001/02 Click here for file

## Figures and Tables

**Table 1:** Distribution of family affluence and parental occupation in 28 European and North American countries in 2001–2002 (%)

		**Family affluence**				**Parental occupation**		

	**N**	**High**	**Medium**	**Low**		**High**	**Medium**	**Low**
Belgium (Flanders)	5720	43.9	21.4	34.7		31.8	42.7	25.6
Canada	3696	35.9	23.5	40.6		47.9	29.7	22.5
Croatia	4207	41.7	20.2	38.0		19.2	31.8	49.0
Czech Republic	4803	40.8	22.2	37.0		43.1	33.2	23.7
Denmark	4036	29.8	42.6	27.6		25.3	25.2	49.5
Estonia	3873	28.2	39.8	32.9		30.4	27.2	42.4
Finland	4738	24.2	42.8	33.0		34.2	41.4	24.4
France	7633	29.3	40.0	30.6		24.7	25.2	50.2
Germany	5080	28.9	39.1	32.1		20.4	40.7	38.9
Greece	3720	37.0	39.1	23.8		29.5	33.2	37.3
Hungary	3879	29.7	36.1	34.2		34.8	45.8	19.4
Ireland	2683	40.4	21.0	38.6		64.3	20.5	15.2
Israel	5053	43.8	17.8	38.4		40.8	34.0	25.1
Italy	4212	35.7	40.1	24.2		20.6	56.1	23.3
Latvia	3007	33.7	34.8	31.5		33.9	25.5	40.6
Malta	1817	32.8	25.8	41.4		29.2	28.1	42.7
Norway	4348	22.8	47.6	29.6		23.0	46.5	30.5
Poland	5935	28.2	37.8	34.1		20.5	31.9	47.6
Portugal	2711	36.3	37.1	26.6		23.2	21.9	54.9
Russia	7005	26.5	39.9	33.5		25.9	45.2	29.0
Scotland	3548	25.1	39.7	35.1		43.6	19.7	36.7
Slovenia	3653	43.9	22.0	34.1		23.0	28.9	48.1
Spain	5620	41.7	20.3	38.0		27.3	18.4	54.3
Sweden	3380	37.7	20.8	41.5		46.4	15.3	38.3
Switzerland	4179	30.6	43.1	26.4		16.2	37.5	46.3
Ukraine	3674	40.4	19.1	40.5		37.6	10.8	51.6
USA	4240	37.7	38.2	24.1		44.4	27.5	28.1
Wales	3144	26.6	43.6	29.9		44.0	18.9	37.1

**Table 2:** The relationships between drunkenness (2 to 3 times or more) and family affluence among 11–15 year olds in Europe and North America in 2001–2002, odds ratios, 95% confidence intervals^1^

	**Boys**						**Girls**					
	
	**Family affluence**						**Family affluence**					
	
	High	Medium		Low			High	Medium		Low		
	
		**OR**	**95% CI**	**OR**	**95% CI**	p^2^		**OR**	**95% CI**	**OR**	**95% CI**	p^2^
Belgium (Flanders)	1.00	0.76	(0.57–1.00)	0.78	(0.61–1.00)	ns	1.00	0.94	(0.68–1.29)	1.00	(0.75–1.33)	ns
Canada	1.00	0.75	(0.56–1.11)	0.78	(0.56–1.10)	ns	1.00	1.09	(0.76–1.56)	1.02	(0.75–1.38)	ns
Croatia	1.00	0.78	(0.57–1.11)	**0.74**	**(0.55–0.98)**	ns	1.00	0.91	(0.60–1.38)	0.89	(0.63–1.25)	ns
Czech Republic	1.00	0.92	(0.68–1.25)	0.87	(0.66–1.15)	ns	1.00	0.84	(0.58–1.21)	0.99	(0.73–1.34)	ns
Denmark	1.00	0.80	(0.61–1.05)	0.99	(0.71–1.38)	ns	1.00	1.07	(0.78–1.46)	0.79	(0.55–1.12)	ns
Estonia	1.00	**0.75**	**(0.57–0.99)**	0.77	(0.57–1.05)	ns	1.00	1.09	(0.77–1.52)	1.16	(0.82–1.65)	ns
Finland	1.00	0.80	(0.60–1.07)	0.97	(0.72–1.32)	ns	1.00	0.79	(0.58–1.07)	1.19	(0.86–1.63)	**
France	1.00	0.84	(0.63–1.12)	1.08	(0.80–1.47)	ns	1.00	1.13	(0.79–1.62)	1.07	(0.73–1.56)	ns
Germany	1.00	**0.70**	**(0.53–0.93)**	0.78	(0.58–1.07)	**	1.00	0.99	(0.73–1.34)	0.93	(0.67–1.30)	ns
Greece	1.00	0.95	(0.68–1.31)	0.69	(0.45–1.05)	ns	1.00	1.07	(0.71–1.62)	1.12	(0.71–1.79)	ns
Hungary	1.00	**0.62**	**(0.44–0.87)**	**0.61**	**(0.42–0.87)**	**	1.00	0.71	(0.49–1.03)	0.99	(0.69–1.42)	ns
Ireland	1.00	0.74	(0.46–1.19)	0.67	(0.43–1.05)	ns	1.00	0.75	(0.47–1.20)	0.82	(0.56–1.20)	ns
Israel	1.00	0.70	(0.48–1.02)	0.76	(0.57–1.05)	ns	1.00	0.76	(0.46–1.27)	0.87	(0.59–1.29)	ns
Italy	1.00	0.76	(0.54–1.07)	0.92	(0.60–1.40)	ns	1.00	0.71	(0.48–1.06)	0.97	(0.61–1.53)	ns
Latvia	1.00	**0.69**	**(0.49–0.96)**	0.71	(0.50–1.04)	*	1.00	0.72	(0.48–1.08)	**0.66**	**(0.44–0.99)**	ns
Malta	1.00	0.83	(0.47–1.46)	0.74	(0.44–1.25)	ns	1.00	1.08	(0.60–1.98)	0.83	(0.45–1.54)	ns
Norway	1.00	0.76	(0.55–1.06)	0.84	(0.58–1.23)	ns	1.00	1.06	(0.75–1.51)	1.12	(0.77–1.65)	ns
Poland	1.00	0.81	(0.63–1.05)	0.80	(0.61–1.05)	ns	1.00	0.75	(0.55–1.04)	1.04	(0.76–1.44)	ns
Portugal	1.00	**0.62**	**(0.43–0.91)**	**0.49**	**(0.30–0.80)**	**	1.00	0.70	(0.43–1.14)	0.76	(0.44–1.30)	ns
Russia	1.00	**0.78**	**(0.63–0.97)**	**0.64**	**(0.51–0.82)**	**	1.00	1.05	(0.82–1.33)	0.85	(0.66–1.09)	ns
Scotland	1.00	**0.64**	**(0.46–0.87)**	0.89	(0.64–1.25)	*	1.00	1.13	(0.79–1.61)	1.11	(0.76–1.62)	ns
Slovenia	1.00	0.73	(0.52–1.01)	0.81	(0.59–1.09)	ns	1.00	1.05	(0.71–1.57)	0.71	(0.48–1.04)	ns
Spain	1.00	0.90	(0.61–1.31)	0.87	(0.62–1.22)	ns	1.00	0.88	(0.59–1.31)	0.97	(0.69–1.35)	ns
Sweden	1.00	**0.45**	**(0.30–0.68)**	**0.43**	**(0.30–0.62)**	***	1.00	1.10	(0.71–1.69)	1.25	(0.87–1.78)	ns
Switzerland	1.00	0.96	(0.71–1.29)	1.07	(0.76–1.51)	ns	1.00	0.90	(0.60–1.34)	1.02	(0.66–1.57)	ns
Ukraine	1.00	1.11	(0.81–1.53)	1.08	(0.83–1.40)	ns	1.00	0.86	(0.62–1.20)	0.91	(0.70–1.18)	ns
USA	1.00	0.81	(0.58–1.12)	0.78	(0.51–1.19)	ns	1.00	1.24	(0.89–1.72)	1.22	(0.82–1.84)	ns
Wales	1.00	1.10	(0.83–1.46)	0.91	(0.66–1.27)	ns	1.00	1.10	(0.78–1.53)	0.88	(0.62–1.27)	ns
All countries	1.00	0.82	(0.77–0.86)	0.80	(0.76–0.85)	***	1.00	1.02	(0.96–1.09)	0.99	(0.94–1.05)	ns

^1 ^logistic regression models. adjusted for age and parental occupation

^2 ^overall effect of the variable in the model

* p < 0,05, ** p < 0,01, *** p < 0,001

**Table 3:** The relationships between drunkenness (2 to 3 times or more) and parental occupation among 11- 15 year olds in Europe and North America in 2001–2002, odds ratios, 95% confidence intervals^1^

	**Boys**						**Girls**					
	
	**Parental occupation**						**Parental occupation**					
	
	High	Medium		Low			High	Medium		Low		
	
		**OR**	**95% CI**	**OR**	**95% CI**	p^2^		**OR**	**95% CI**	**OR**	**95% CI**	p^2^
Belgium (Flanders)	1.00	1.24	(0.96–1.61)	**1.40**	**(1.05–1.86)**	ns	1.00	**1.35**	**(1.00–1.81)**	**1.69**	**(1.21–2.36)**	**
Canada	1.00	1.18	(0.84–1.65)	1.09	(0.75–1.59)	ns	1.00	1.30	(0.96–1.75)	1.16	(0.83–1.63)	ns
Croatia	1.00	1.25	(0.87–1.80)	**1.44**	**(1.03–2.03)**	ns	1.00	1.01	(0.67–1.53)	0.80	(0.53–1.20)	ns
Czech Republic	1.00	0.95	(0.72–1.26)	**1.60**	**(1.18–2.16)**	**	1.00	1.18	(0.86–1.62)	1.36	(0.98–1.88)	ns
Denmark	1.00	1.33	(0.96–1.83)	1.29	(0.96–1.73)	ns	1.00	1.21	(0.86–1.69)	1.05	(0.77–1.44)	ns
Estonia	1.00	0.88	(0.65–1.18)	1.09	(0.83–1.43)	ns	1.00	0.85	(0.61–1.19)	1.00	(0.74–1.36)	ns
Finland	1.00	**1.34**	**(1.03–1.75)**	1.20	(0.89–1.63)	ns	1.00	**1.35**	**(1.04–1.74)**	**1.37**	**(1.02–1.85)**	**
France	1.00	0.73	(0.53–1.01)	0.76	(0.57–1.01)	ns	1.00	**0.44**	**(0.29–0.67)**	0.76	(0.55–1.05)	**
Germany	1.00	1.12	(0.83–1.52)	0.91	(0.65–1.27)	ns	1.00	1.31	(0.95–1.81)	1.08	(0.76–1.55)	ns
Greece	1.00	1.02	(0.71–1.46)	1.04	(0.72–1.50)	ns	1.00	1.04	(0.68–1.60)	0.83	(0.52–1.31)	ns
Hungary	1.00	**1.41**	**(1.04–1.91)**	1.55	(0.98–2.48)	*	1.00	1.13	(0.81–1.56)	1.05	(0.70–1.60)	ns
Ireland	1.00	1.03	(0.62–1.70)	1.63	(0.97–2.76)	ns	1.00	**1.61**	**(1.08–2.40)**	1.45	(0.89–2.36)	*
Israel	1.00	1.04	(0.77–1.40)	0.85	(0.59–1.24)	ns	1.00	0.75	(0.50–1.12)	0.68	(0.43–1.07)	ns
Italy	1.00	1.33	(0.87–2.03)	1.15	(0.72–1.84)	ns	1.00	0.83	(0.53–1.30)	1.11	(0.67–1.84)	ns
Latvia	1.00	0.77	(0.53–1.12)	0.81	(0.58–1.13)	ns	1.00	1.12	(0.74–1.69)	1.17	(0.78–1.74)	ns
Malta	1.00	0.66	(0.36–1.22)	0.89	(0.53–1.48)	ns	1.00	1.16	(0.64–2.11)	0.80	(0.42–1.50)	ns
Norway	1.00	1.05	(0.75–1.48)	1.29	(0.89–1.87)	ns	1.00	0.92	(0.65–1.29)	**1.52**	**(1.05–2.19)**	**
Poland	1.00	**1.43**	**(1.09–1.90)**	1.29	(0.97–1.70)	*	1.00	1.03	(0.74–1.42)	**0.69**	**(0.49–0.96)**	*
Portugal	1.00	1.11	(0.69–1.80)	1.43	(0.94–2.17)	ns	1.00	1.48	(0.80–2.75)	1.60	(0.91–2.79)	ns
Russia	1.00	1.22	(0.98–1.53)	**1.46**	**(1.13–1.87)**	*	1.00	0.93	(0.74–1.17)	1.27	(0.99–1.63)	*
Scotland	1.00	1.14	(0.81–1.60)	1.14	(0.85–1.54)	ns	1.00	0.93	(0.65–1.34)	1.34	(0.99–1.82)	ns
Slovenia	1.00	1.40	(0.99–1.99)	**1.57**	**(1.12–2.19)**	*	1.00	1.42	(0.92–2.19)	1.11	(0.72–1.71)	ns
Spain	1.00	1.16	(0.76–1.75)	1.31	(0.93–1.85)		1.00	0.98	(0.66–1.47)	0.75	(0.54–1.05)	ns
Sweden	1.00	**1.89**	**(1.21–2.96)**	**1.57**	**(1.11–2.22)**	**	1.00	1.01	(0.65–1.57)	1.21	(0.86–1.71)	ns
Switzerland	1.00	0.96	(0.66–1.40)	1.04	(0.71–1.51)	ns	1.00	1.11	(0.70–1.77)	0.82	(0.51–1.33)	ns
Ukraine	1.00	1.12	(0.73–1.73)	1.04	(0.82–1.34)	ns	1.00	0.84	(0.57–1.24)	0.88	(0.68–1.13)	ns
USA	1.00	1.22	(0.85–1.73)	1.25	(0.85–1.84)	ns	1.00	0.91	(0.64–1.29)	1.00	(0.69–1.43)	ns
Wales	1.00	**1.45**	**(1.04–2.02)**	1.24	(0.94–1.62)	ns	1.00	1.09	(0.77–1.53)	1.21	(0.90–1.63)	ns
All countries	1.00	**1.08**	**(1.02–1.14)**	**1.15**	**(1.09–1.21)**	***	1.00	1.01	(0.95–1.07)	1.02	(0.96–1.08)	ns

^1 ^logistic regression models. adjusted for age and family affluence

^2 ^overall effect of the variable in the model

* p < 0,05, ** p < 0,01, *** p < 0,001
