# Supplementary material for: The relationship between parental socio-economic status and episodes of drunkenness among adolescents: findings from a cross-national survey
Source: BMC Public Health. 2006 Nov 28;6:289. doi: 10.1186/1471-2458-6-289 (PMC1693920; doi:10.1186/1471-2458-6-289)
Supplement: Additional file 1 — Appendix 1: Information on level of consent and ethical approval in the HBSC study 2001/02 [file 1471-2458-6-289-S1.doc]

### Additional file 1 – Level of consent and ethical approval in HBSC.doc

Appendix 1: Information on level of consent and ethical approval in the HBSC study 2001/02 ^1^

|  | **Level of consent in school** | | | | |  |  |
| --- | --- | --- | --- | --- | --- | --- | --- |
|  | **School level (headmaster/ teacher)** | **Parental level** | | **Student level** | | **Ethical approval** | **Name of review committee** |
| **Belgium (Flanders)** | informed consent | no consent | | passive consent | | yes | Ethical committee of the Ghent University Hospital |
| **Canada** | informed consent | informed consent | | passive consent | | yes | Queen's University General Research Ethics Board |
| **Croatia** | informed consent | informed/passive  (depends on school) | | informed consent | | not required/  applicable at the time | / |
| **Czech Republic** | no consent | no consent | | no consent | | not required/  applicable at the time | / |
| **Denmark** | informed consent | informed consent from parents representatives | | informed consent  from students representatives | | not required/  applicable at the time | / |
| **Estonia** | informed consent | informed consent | | informed consent | | not required/  applicable at the time | / |
| **Finland** | informed consent | no consent | | passive consent | | yes | Ethical board of research of the University of Jyväskylä |
| **France** | no consent | passive consent | | passive consent | | not required/  applicable at the time | / |
| **Germany** | informed consent | informed consent | | passive consent | | yes | Data protection commissioner of the federal states of Berlin, Hesse, Saxony,and Northrhine-Westphalia |
| **Greece** | informed consent | passive consent | | informed consent | | yes | National Pedagogic Institute |
| **Hungary** | informed consent | passive consent | | informed consent | | yes | Scientific and Research Ethical Committee of the Medical Research Council of the Hungarian Ministry of Health |
| **Ireland** | informed consent | informed/passive (depends on school) | | passive consent | | yes | The Research Ethics Committee of the Faculty of Public Health Medicine |
| **Israel** | passive consent | passive consent | | Informed consent. | | yes- | Ministry of Education’s chief scientist. |
| **Italy** | informed consent | passive consent | | Informed consent | | yes | Ministry of Education Undersecretary Office of the Ministry |
| **Latvia** | informed consent | no consent | | informed consent | | not required/  applicable at the time | / |
| **Malta** | no information available | | | | | |  |
| **Norway** | informed consent | passive consent | | informed consent | | yes | Ombudsman for privacy protection, Norwegian Social Science Data Services |
| **Poland** | informed consent | passive consent | | informed consent | | not required/  applicable at the time | / |
| **Portugal** | informed consent | passive consent | | passive consent | | yes | University committee  (Fundação para a Ciencia e Tecnologia; Faculdade de Motricidade Humana) |
| **Russia** | no information available | | | | | |  |
| **Scotland** | informed consent | | passive consent | | passive consent | not required/  applicable at the time | / |
| **Slovenia** | informed consent | | passive consent | | passive consent | not required/  applicable at the time | / |
| **Spain** | informed consent | | passive consent | | passive consent | yes | Ethical committee of Experimentation of the University of Seville |
| **Sweden** | informed consent | | passive consent | | informed consent | not required/  applicable at the time | / |
| **Switzerland** | passive consent | | no consent | | passive consent | not required/  applicable at the time | / |
| **Ukraine** | informed consent | | no consent | | informed consent | yes | Ethical Committee of the Sociological Association of Ukraine |
| **USA** | informed consent | | informed/passive (depends on school) | | informed/passive (depends on school) | Yes | National Institute of Child Health and Human Development Institutional Review Board |
| **Wales** | informed consent | | passive consent | | passive consent | not required/  applicable at the time | / |

^1^ Information provided by the principal investigator of each country. The list of principal investigators at the time of the survey can be found in the acknowledgments.
